# Vertebral Primary Bone Lesions: Review of Management Options

**DOI:** 10.3390/curroncol30030232

**Published:** 2023-03-04

**Authors:** Anjalika Chalamgari, Daisy Valle, Xuban Palau Villarreal, Marco Foreman, Annika Liu, Aashay Patel, Akanksha Dave, Brandon Lucke-Wold

**Affiliations:** Department of Neurosurgery, University of Florida College of Medicine, Gainesville, FL 32601, USA

**Keywords:** vertebral primary bone lesions, surgical interventions, nonsurgical interventions, primary malignant vertebral lesions, primary benign lesions

## Abstract

The assessment and treatment of vertebral primary bone lesions continue to pose a unique yet significant challenge. Indeed, there exists little in the literature in the way of compiling and overviewing the various types of vertebral lesions, which can often have complicated intervention strategies. Given the severe consequences of mismanaged vertebral bone tumors—including the extreme loss of motor function—it is clear that such an overview of spinal lesion care is needed. Thus, in the following paper, we aim to address the assessment of various vertebral primary bone lesions, outlining the relevant nonsurgical and surgical interventional methods. We describe examples of primary benign and malignant tumors, comparing and contrasting their differences. We also highlight emerging treatments and approaches for these tumors, like cryoablation and stereotactic body radiation therapy. Ultimately, we aim to emphasize the need for further guidelines in regard to correlating lesion type with proper therapy, underscoring the innate diversity of vertebral primary bone lesions in the literature.

## 1. Introduction

Vertebral primary bone lesions have long presented a challenge to spinal care specialists. Not only is there a wide variety of spinal lesions described in the literature, the treatment of these tumors, benign or otherwise, is often complex and complicated by factors such as neural compression [1]. Vertebral lesions are also frequently found incidentally when the patient presents with back pain, weakness, or myelopathy, and if management is delayed, vertebral lesions can lead to a complete loss of sensory and motor function [2]. A thorough, comprehensive understanding of the assessment and treatment of these lesions is needed.

There are several classifications for vertebral bone lesions. By itself, vertebral bone lesions are a form of spinal lesion that only affects the osseous portion; thus, they are referred to as spinal osseous lesions, spinal neoplasms, vertebral lesions, or vertebral tumors [3]. A vertebral primary bone lesion is a general term describing any abnormal change to the bone originating from disease or injury to healthy bone. In this context, primary is an oncological term that indicates the lesion originates from the bone, whereas secondary lesions are when the lesion metastasizes to the bone from another region of the body [4]. When cells in the bone undergo uncontrolled growth, these lesions are referred to as bone tumors, and when the abnormal tissue closely resembles the healthy bone structure, the lesion is considered benign [5]. Examples of benign lesions include hemangiomas, lipomas, sclerosis, aneurysmal bone cysts, osteoid osteomas, and osteoblastomas [6]. Malignant lesions are similar to bone tumors but demonstrate a growth capable of metastasis [7]. Malignant vertebral lesions include chondrosarcoma, chordoma, Ewing’s sarcoma, neuroblastoma, and osteosarcoma [8]. Classification of a lesion depends on the spinal level, location of the lesion on the vertebra, layer of bone affected, number of lesions, and morphology. Typically, since lesion type determines treatment, correct identification of the lesion can have a major role in clinical outcomes [9].

Interventions for vertebral lesions are divided into surgical excision and non-surgical medical management. In recent years, low-grade tumors have even been approached with more innovative techniques, like phenol, cryosurgery, or argon lasering [10]. Such targeted therapies can be less invasive and speed the recovery process. A plethora of interventions exists in the context of vertebral tumors. Yet, while much of the literature describes spinal lesions and corrective measures for specific tumors, there is a paucity of studies available that discuss these different lesion types and explain general guidelines for their treatment [1,11,12]. Most reviews on vertebral lesions are based on a series of case reports, and indeed there is little literature generally overviewing these tumors. Thus, this paper attempts to take a more holistic approach, and we describe the assessment, non-surgical treatment, and surgical treatment of various vertebral bone lesions, categorized as either primary benign, malignant, or metastatic.

## 2. Assessment of Patients with Vertebral Lesions

### 2.1. Incidence Rates

**Figure 1:**
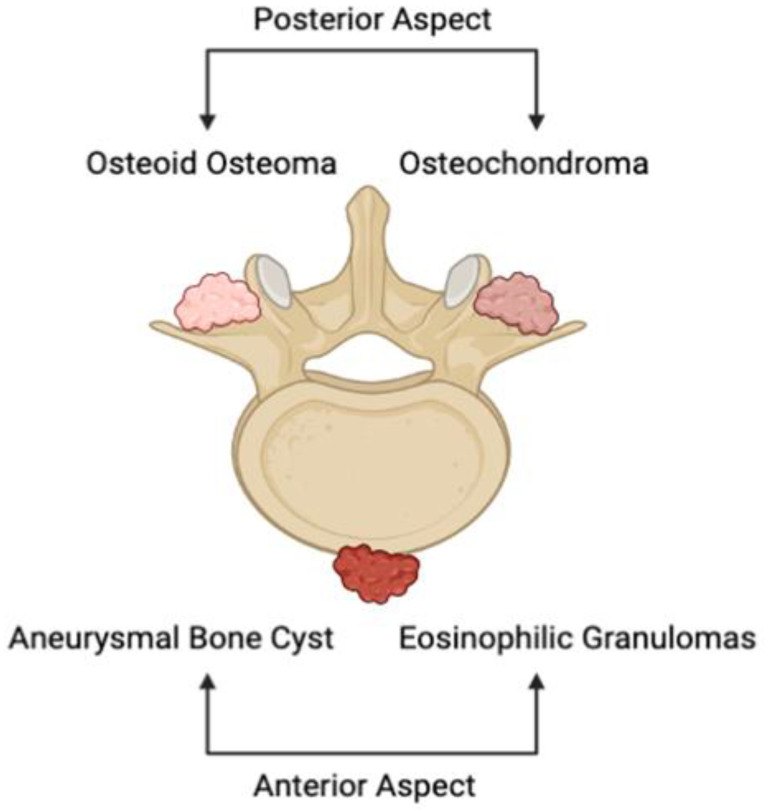
Illustrative depiction of the anatomical location of common primary osseous lesions of the vertebrae. Aneurysmal bone cysts and eosinophilic granulomas depict prevalent primary vertebral tumors of the anterior portion of the spine, while osteoid osteomas and osteochondromas represent lesions that represent those witnessed on the posterior portion of the vertebrae.

Primary osseous lesions of the spine encompass a divergent series of malignant and benign tumors. The majority, which are non-cancerous, can be primarily found in the anterior portion of the vertebral body (Figure 1) [13]. Examples of benign tumors of the vertebrae include aneurysmal bone cysts, eosinophilic granulomas, and hemangiomas. Others, notably osteoid osteomas and osteochondromas, are more commonly witnessed in the posterior elements of the vertebral body (Figure 1). While the growths in question are considerably less prevalent than the occurrence of metastases, primary bone tumors of the vertebrae constitute the most prevalent category of primary osseous lesions [14]. Nevertheless, the true incidence of primary tumors of the spine is unclear due to their infrequency and the fact that the majority of these growths present asymptomatically [15]. Primary vertebral tumors are seen to comprise approximately 0.2% of all cancers that are newly identified each year, further accounting for 5% of the overarching bone tumors category [16]. A thorough inspection of these incidence rates has led to the identification of an emergent trend: more than 90% of lesions can be found to be benign when a person is in their first decade of life, around 50% when they are in the fourth decade, and fewer than 10% when they are in the seventh decade [17]. Of those found cancerous, the rates of recurrence and mortality are elevated up to 48% and 58%, respectively [14]. In spite of the fact that these spinal growths are largely heterogeneous, it is still possible to arrive at an appropriate differential diagnosis and treatment through the methodical consideration of a patient’s full history of the present illness and radiological pattern.

### 2.2. Clinical Considerations

When examining individuals with spine tumors, the history of present and prospective lesions, as well as carcinogen exposure, must be assessed. Previously, accounts of oncological patients that harbored spinal metastases years after effective treatment have been reported [15]. As such, it is crucial to consider that benign skeletal lesions in any region of the body may culminate in spine metastasis following malignant or sarcomatous transformation [18]. In individuals with vertebral growths, pain is perhaps the most prevalent and predominant symptom [19,20]. As seen with the vast majority of skeletal system tumors, patients may attribute their pain to a factual or hypothesized traumatic event that occurred in the recent past [15]. Occasionally, this situation implies a pathological fracture resulting from the collapse of a vertebral body due to physical trauma [21]. In the same realm, the most consistent characteristic of primary osseous lesions is discomfort that develops gradually, intensifies with time, and persists at night, ultimately impacting sleeping behavior [20]. Acute pain that develops in the absence of trauma in a patient with no preceding symptoms should also be regarded as a pathological fracture in such a clinical context [15].

### 2.3. Imaging Considerations

Despite the fact that the majority of presentations of low back pain heal with conservative therapy, radiographs are frequently employed as an initial preventive screening. The quick availability and inexpensive cost of radiographs prove useful when assessing for fractures, vertebral degeneration, and bone density [22,23]. For patients presenting with more severe symptoms, suspected underlying infection, or implications of the cauda equina, CT and MRI are the superior imaging modalities employed [24]. CT allows for better visualization of the tumor microenvironment, even to a greater extent than MRI [19]. Furthermore, it enhances the visibility of pathological degenerative developments and fractures, especially in the posterior components [22]. On the other hand, MRIs are more frequently utilized in the presence of secondary neurological symptoms, providing increased soft tissue contrast to CT to characterize intervertebral disks, spinal bone marrow, and contents of the vertebral foramen [19,23]. Together, however, CT and MRI are utilized in unison to depict a full picture of the issue.

**Figure 2:**
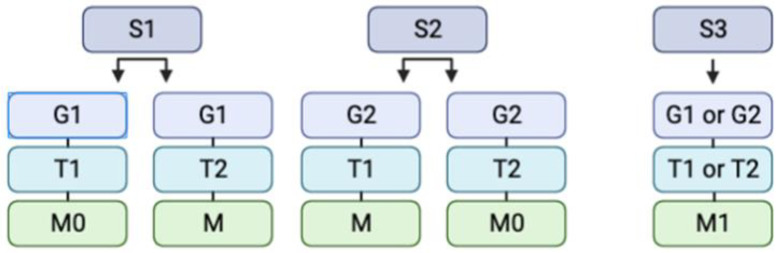
Illustrative depiction of a simplified Enneking classification system, which categorizes tumors into stage, grade, site, and presence of metastasis. The abbreviations are as follows: S = Stage (1, 2, or 3), G = Grade (1 or 2), T = Site (1 or 2), and M = Metastasis (0 or 1). S1 indicates that the growth is inactive, asymptomatic, developing slowly or not at all, and has a true capsule. S2 indicates that the lesion is mildly symptomatic, developing with an outline, and displays a minimal risk of recurrence. S3 indicates that the tumor is expanding fast, the capsule has been broken or is completely missing, there is invasion of surrounding tissues, and the recurrence rate is significant. G1 and G2 are indicative of low and high grades, accordingly. The T1 and T2 indicate intraosseous and extraosseous growth, respectively. Finally, M0 indicates the absence of metastasis and M1 the presence of metastasis.

On radiographs, lesions are often classified utilizing the Enneking classification system, which aids in the assignment of tumors into two categories: benign and malignant (Figure 2) [24,25]. In accordance with the Enneking classification system, non-cancerous tumors are further divided into the following categories: S1 indicates that the growth is inactive, asymptomatic, developing slowly or not at all, and has a true capsule [26]. S2 indicates that the lesion is mildly symptomatic, developing with an outline, and displays a minimal risk of recurrence [25]. Finally, S3 indicates that the tumor is expanding fast, the capsule has been broken or is completely missing, there is an invasion of surrounding tissues, and the recurrence rate is significant [26]. The localization of the tumors is delineated through the use of T1 and T2 designations, defining intraosseous or extraosseous growth accordingly. It should not go without consideration that the presence of metastasis is noted as well. Similarly, lesions may be categorized additionally according to their grade: 1 and 2 are indicative of a low and high grade, respectively [27]. Ultimately, however, descriptive words help radiologists and neurosurgeons distinguish growths. For instance, hemangiomas can be described as having a “salt and pepper” look, with a high signal intensity, and exhibiting considerable contrast [28,29]. Osteoid osteomas, in comparison, may reveal a radiolucent tumor with surrounding sclerosis, bearing a resemblance to a “sunny-side-up egg” [30,31,32]. A full list of the salient radiographic findings for common benign and malignant bony tumors is provided in Table 1.

## 3. Treatment of Patients with Vertebral Lesions

### 3.1. Primary Benign Tumors

Primary benign vertebral lesions are often asymptomatic and undetected [33], but when they progress and do become noticed, they require surgical excision. Treatments vary between types of benign vertebral lesions, but approaches generally depend on the risk of recurrence, surgical complications, complex reconstruction, functional deficits, and worsening of the patient’s health condition. In most cases, intralesional curettage, bone grafting, and synthetic bone substitutes are used to maintain the structural integrity and stability of the vertebra and spine [34]. To prevent the recurrence of the vertebral lesion, adjuvant measures like phenol instillation or cryotherapy may be used post-surgically. Small or slow-developing lesions may only require corticoid injections or percutaneous vertebroplasty to eradicate the tumor. Novel therapies for fibrous dysplasia and giant cell tumors are using less invasive [35] and more systemic therapies with bisphosphonates and denosumab, respectively [36].

Recently, some benign tumors have been treated by the administration of intravenous bisphosphonate, which inhibits the bone resorption mediated by osteoclasts. More specifically, Pamidronate and Zoledronic acid are used for their anti-tumor features and ability to reduce the bone tumor burden, prevent metastasis, and inhibit the progression of a bone lesion [37]. Moreover, excision or curettage aimed toward removing the benign tumor is the most common surgical treatment. Complete tumor removal attempts to preserve as much bone as possible and must be performed through an acceptable cortical window that ensures proper visualization of the entire lesion [38]. Aggressive local tumor removal along with mechanical, thermal, and/or chemical adjuvant therapy depicts extended intralesional curettage. This is administered as a method of extending the tumor kill zone far beyond the limit of mechanical curettage [39]. A cortical window is obtained to allow for visualization of the bone lesion, followed by a mechanical adjuvant of a high-speed burr to increase the borders of the lesion area.

The use of curettage with adjuvants is reported to be associated with low local recurrence rates ranging from 12–34% [40]. Other types of adjuvant therapies, such as argon bean, hydrogen peroxide, liquid nitrogen, and alcohol, are being evaluated but have yet to demonstrate a significant impact [41]. Hydrogen peroxide has been examined when used as a local adjuvant to potentially kill the remaining tumor cells that are left in the tumor cavity after curettage [42]. The use of hydrogen peroxide as a local adjuvant should be explored as a more permanent treatment procedure due to its demonstrated ability to induce apoptosis and reduce recurrence rates [43]. When categorized as a benign aggressive or low-grade malignant bone tumor, cryotherapy with liquid nitrogen is as effective as wide excision [44]. More effectively than phenol and cement, cryosurgery produces cell necrosis of up to 2 cm from the bone surface [45]. The surgical procedure entails exposure, curettage, margin expansion, cryosurgery, bone cement reconstruction, and subchondral bone graft to protect from future impact potentially leading to fracture [44]. Deventer et al. report the overall recurrence rate of chondroblastoma with intralesional curettage as surgical treatment to be 39.5% [46]. In this retrospective study, 7% only showed intraoperative complications from the remaining fragment of bone cement with a distribution of types of adjuvants used: 44% hydrogen peroxide, 46% bone substitute, 7% autogenous bone graft, and 5% bone cement.

**Figure 3:**
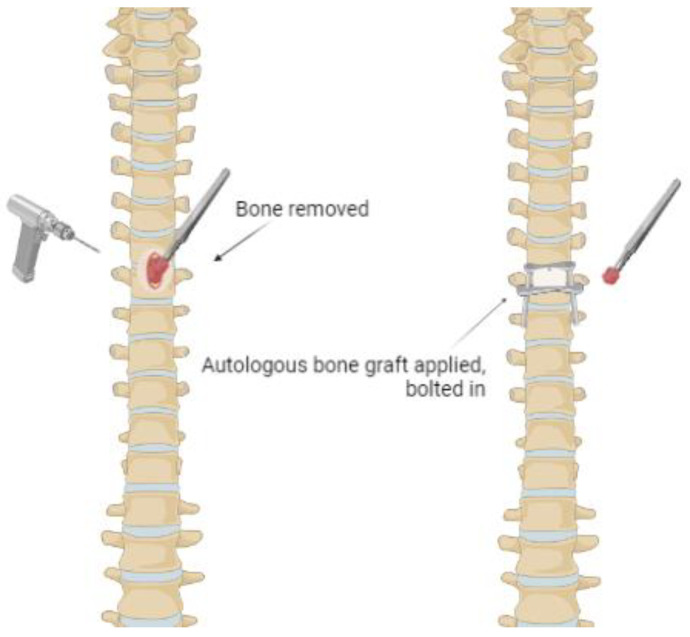
Autogenous bone graft from a primary benign tumor in the vertebrae.

The gold standard of treatment for primary benign tumors is autogenous bone grafts (Figure 3). The components of osteogenic stem cells, growth factors and matrix ensure proper integration and healing of the graft [47]. Studies report vascularized fibular grafts to be more quickly incorporated into the lesion, which can reduce surgical complications [48]. In addition, when comparing vascularized fibular grafts to non-vascularized grafts, vascularized grafts prove to be superior in the treatment of aggressive benign bone tumors. The disadvantages of bone grafts are surgical morbidity at the donor site, the risk of immune rejection, disease transmission, and infection [49]. For the treatment of benign bone tumors and tumor-like bone lesions that are characterized by a large bone defect, allogeneic cortical support as a reconstructive technique provides high resistance, easy fixation, fracture healing and prevention of deformity [50]. Even though this reconstructive technique provides remodeling of the site, it may occur slowly or even remain incomplete. If necessary, allogenic cancellous bone grafts can be implemented with adjuvants such as alcohol and phenol.

Seeking the creation of a structural framework environment that is favorable for cell function and the formation of new bone is the purpose of a synthetic bone substitute. Complete integration of the synthetic bone graft into the host is achieved through the substitute’s ability for osteoinductive growth with undifferentiated primitive mesenchymal cells [51]. Injections of autologous bone marrow with multipotent stem cells, bone morphogenic proteins and growth factors ensure osteogenesis and an osteoconductive environment. Some of the primary ceramic components of the graft are osteoconductive to reconstructive and include calcium phosphate, hydroxyapatite, tricalcium phosphate, and calcium sulfate [52].

### 3.2. Non-Surgical Treatment for Primary Malignant Tumors

Primary malignant tumors, unlike primary benign tumors, can be treated either surgically or non-surgically. In the non-surgical context, radiotherapy (RT) of the malignant tumor has long been considered the gold standard for care [53]. The goal of RT is to destroy the tumor cells while simultaneously avoiding damage to the normal cells [54]. Importantly, although RT can act as an analgesic, it does not reverse secondary bone or tissue destruction [55]. The use of RT also warrants other clinical considerations. One study by Ghogawala et al. found, for example, that patients who had surgery after RT had increased rates of post-surgical complications [56]. However, this study was conducted in the context of metastatic spine disease, so more work is needed to establish whether these results are translatable to primary tumors.

Despite its potential drawbacks, RT has shown to be as effective at mitigating relapse and improving survival relative to surgical methods [57]. Long-term benefits of RT, including a reduction in vertebral fracture incidence, have also been demonstrated [58]. Yet, while it has been validated in some of the most common primary benign tumors, including Ewing’s sarcoma and hemangiomas, radiotherapy remains understudied in rarer lesions [59,60].

Typically, chemotherapy is incorporated into most RT treatment regimens. One study suggested that chemotherapy significantly increased the survival rate of patients by almost 70% and that relapse was avoided much more frequently in those receiving chemotherapy than in those who were not [55]. In patients with Ewing’s sarcoma, Rosen et al. found that patients who received chemotherapy in combination with RT had a cancer-free survival rate of almost 76%, which was similar to the 82% survival rate of those receiving surgery [61].

Aside from chemotherapy and radiotherapy, embolization occludes feeding arteries supplying the aneurysmal and giant cell tumors to reduce tumor rise, reduce compression, and restore neurological function [62]. Preoperative embolization de-vascularizing the spinal tumor prior to resection is a minimally invasive procedure that helps reduce intraoperative bleeding and a number of necessary transfusions [63]. Preoperative embolization also allows for more radical resection of the vertebral lesion [64]. However, there remains a risk for postoperative paralysis from the permanent occlusion of a feeding artery to the spinal cord after embolization [65].

**Figure 4:**
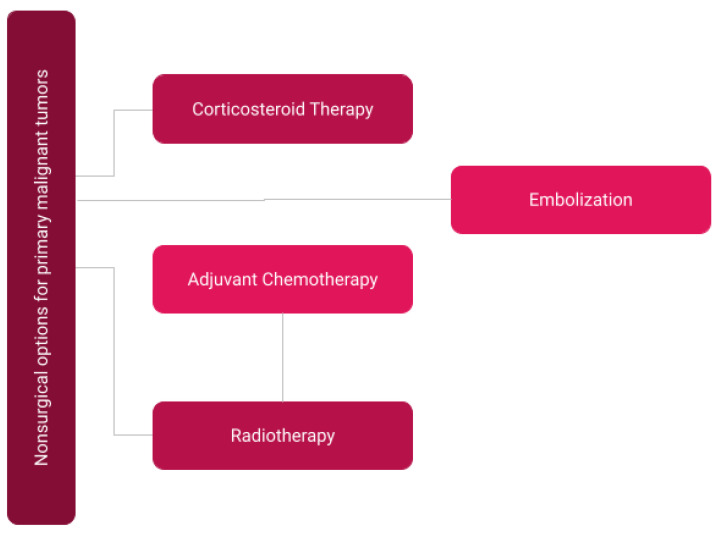
Overview of non-surgical considerations for primary malignant tumors in patients.

Severe pain during metastases, surgical recovery, and therapy significantly affects the quality of life of malignant primary spine tumor patients, thus making multimodal pain management a major aspect of their nonsurgical treatment [66,67]. In cases of mild pain, cyclooxygenase-inhibiting NSAIDs are administered to reduce pain caused by inflammation. In contrast, cases of severe pain commonly involve systemic management by oral, transcutaneous, or intravenous opioids. Systemic dexamethasone is sometimes used to prevent neurological deficits from spinal cord compression by subduing inflammation and swelling [68]. Local anesthetics, such as intrathecal or epidural morphine, can target pain specific to the lesion site. Radiopharmaceuticals strontium-89 and samarium-153, when conjugated to pyrophosphate, are specific to managing metastatic bone pain; they advantageously maximize concentration in vertebral lesions while minimizing systemic circulation and risks of toxicity [55]. Considerations for nonsurgical primary malignant tumor therapy are depicted in Figure 4.

### 3.3. Surgical Interventions for Primary Malignant Tumors

#### 3.3.1. Surgical Determination

Overall, the primary objective of surgical intervention is to restore the quality of life to patients via the mitigation of pain symptoms and neurologic functional deficits associated with the tumor [68]. As such, surgeons must first determine if a patient is a suitable candidate for a surgical route of intervention. Numerous factors play a significant role in the potential outcome following surgery, including the current and expected quality of life post-operation, tumor load, and life expectancy [27]. Additionally, as aforementioned, there have been multiple attempts at devising a robust prognostic scoring system to help assist surgeon decision-making for the treatment of malignant vertebral lesions [27,69]. The Tomita prognostic score summarizes its score by weighing the presence and treatability of primary tumors, visceral metastases, and bone metastases. An alternative, the Tokuhashi scoring system, includes additional metrics for its calculation, including the primary location of cancer, Karnofsky performance status, paralysis, and extraspinal bone metastases, in addition to the three components listed in the Tomita score [70]. These scores are often considered concurrently and generally recommend similar treatment options based on the determined prognostic category [69,71,72]. For patients with extremely poor prognoses, generally conservative therapies are recommended, while patients with good or acceptable prognoses are considered candidates for surgical excision and stabilization if needed. A retrospective study on patients with spinal metastases demonstrated that these scores are accurate in differentiating patients with poor prognoses—and thus not good surgical candidates—from patients with generally acceptable prognoses who could potentially benefit from surgery [71]. More work is needed to determine whether this could be applied to primary tumors.

#### 3.3.2. Direct Surgical Decompression and Stabilization

**Figure 5:**
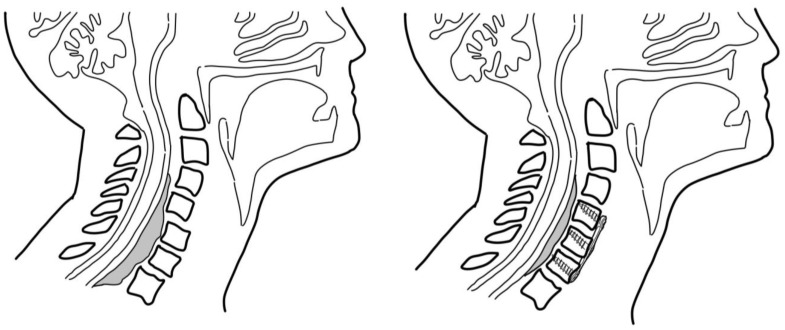
Depiction of pre and post-intervention anterior cervical fusion for extradural vertebral lesions.

Primarily, the most well-documented and understood surgical procedure indicated for vertebral lesions that encroach upon the spinal canal is direct surgical decompression [73,74,75,76]. These procedures achieve a two-fold goal, firstly removing any source of tumor compression on the spinal cord and, secondly, excising tumoral tissue to reduce its total volume in the spine [77]. The relative position of the tumor (anterior, lateral, or posterior) dictates the respectively located approach taken for this procedure, aside from anterior tumors in the thoracic and lumbar spine, which possibly could warrant a transversectomy or anterior vertebrectomy [74,78]. A schematic of pre- and post-intervention anterior cervical fusion is provided in Figure 5. For posteriorly located tumors, a standard decompressive laminectomy procedure is determined to be sufficient.

**Figure 6:**
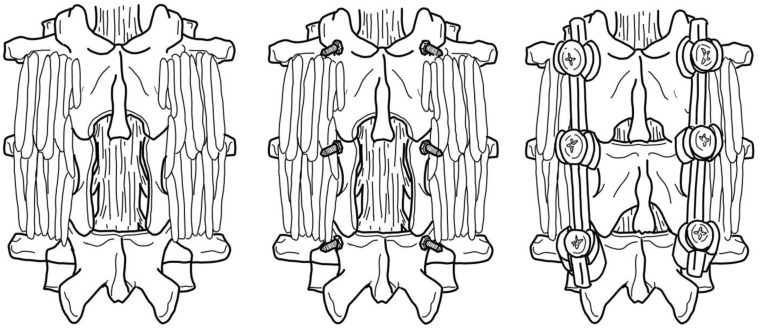
Schematic of laminectomy protocol. Added metal instrumentation enhances spinal stability and improves outcomes following surgery [84].

These techniques are generally applied for a variety of spinal tumors, including multiple myeloma associated with neurological deficits or metastases derived from a primary extraspinal tumor [77,78,79,80,81,82,83]. It should be noted that the majority of cases that do necessitate surgical intervention are malignant cases, as opposed to benign cases, since malignancies typically will encroach into the spinal canal and produce compression upon spinal nerves. However, special considerations must be made depending on the tumor type and derivation. For example, it is recommended that most decompressive surgeries for vertebral body lesions are immediately followed with metal instrumentation to provide additional spinal stability, as this has been highly associated with improved pain reduction and functional outcomes in patients [84,85,86]. A depiction of laminectomy with the inclusion of metal instrumentation for stability is provided in Figure 6. Furthermore, conditions such as multiple myeloma may not necessarily need decompressive intervention if it is sufficiently determined that there is an absence of neurological deficits [82]. Instead, the primary surgical goal is to correct damage caused to vertebral bodies and alleviate related pain symptoms. This can be achieved via multiple approaches, depending on the location and severity of damage [87]. For example, anterior damage to the vertebral body warrants a ceramic or metal endoprosthesis for vertebral body replacement [82,88]. For more posterior, uncomplicated vertebral damage, balloon kyphoplasty and vertebroplasty are used to reinforce the remaining bone with cement [89,90].

The application of recombinant human bone morphogenic proteins (BMP) in such decompressive spinal surgeries has recently captured clinical interest. BMPs are growth factors believed to induce both bone and cartilage formation [91]. Evidence indicates that these factors can significantly enhance bone deposition at needed fusion sites following lumbar decompression [92]. This is supported by similar studies that find their use both safe and efficacious in the context of spinal surgery [93]. Though the available data is limited, the inclusion of BMPs should be an important consideration in any decompressive surgery.

#### 3.3.3. Minimally Invasive Surgical Alternatives

As an alternative approach to conventional surgical intervention, radiosurgery has been gaining popularity in its use to treat spinal lesions [73,94]. Historically, when laminectomy procedures were deemed to be ineffective in reducing pain and restoring the functional status of patients, conventional external beam radiation therapy (cEBRT) was chosen to be the standard of care. Recent advancements in radiation technology have led to the development of spinal stereotactic radiosurgery, which allows for localized delivery of high radiation doses to the target tissue while maintaining a steep drop-off of radiation to surrounding tissues [94,95]. Various studies demonstrate that spinal stereotactic radiosurgery is effective for treating solid metastatic tumors of the spine [96,97]. While less research has been done in the field of primary malignant tumors, one study by Chang et al. successfully adopted spinal stereotactic radiosurgery in that context, demonstrating that the results of previous work could be translatable [98]. Additionally, laser technology such as the argon surgical laser is used as a micro-neurosurgical alternative approach. This micro-laser approach allows for the fine-tuned modulation of laser power through aqueous media, which can be leveraged for the removal of small, deeply located intraspinal tumors [99,100].

## 4. Emerging Interventions

As aforementioned, the standard treatment for complex benign and malignant vertebral primary bone lesions is the surgical intralesional excision/curettage, decompression, and stabilization of the affected area with subsequent bone grafting, namely with autogenous cancellous bone grafts. Particularly, autogenous grafts provide the combination of osteoconduction, osteoinduction, and osteogenic cells that are required for bone regeneration and, ultimately, the capacity to bolster the structural integrity and stability of the surrounding bone and joint [34]. Although recent advancements in bone graft substitutes, including allografts, ceramics, demineralized bone matrix, bone marrow and synthetic composites, have presented similar outcomes to autogenous cancellous grafts, there are numerous shortcomings for each [101]. Principally, none of these alternatives provide a composite of the three requirements for bone regeneration needed for successful bone grafting, and they present with bone quantity limitations and donor-site morbidity. Thus, autogenous grafts remain the gold standard. Further, a wide excision approach, which entails the removal of the tumor along with a capsule of healthy tissue around it, has been reported consistently across the literature for its purported outcome of lower recurrence and mortality rates [102,103]. In other words, surgical intervention necessitates significant excision margins and, subsequently, the replacement with an equally weighty bone graft—two aspects of current treatment that have yet to be circumvented by modern medicine.

As a result, the focus of emerging treatments in the context of vertebral primary bone lesions has been primarily aimed at the employment of adjuvant therapies and other non-invasive measures. Traditionally, adjuvant therapies such as bone cement, phenol, and hydrogen peroxide have been used with the aim of eradicating microscopic infiltrates by mechanical, thermal, or chemical means [49]. Particularly, adjuvant therapy allows surgeons to salvage surrounding healthy bone and tissue while lowering rates of recurrence [104].

The most recent development of such adjuvant interventions can be found in the use of radiotherapy in the treatment of both neoplastic and nonneoplastic processes, such as metastatic multiple myeloma and hemangiomas, respectively [105]. Specifically, a novel treatment modality known as stereotactic body radiation therapy (SBRT) involves the delivery of ultra-high radiation to a small target volume with impressively tight margins [106]. This fact is significant as it confers SBRT an advantage over traditional open surgical techniques by allowing the treatment of tumors close to the spinal cord or at sites of retreatment while serving as a minimally invasive substitute [107]. Moreover, when compared to other minimally invasive counterparts such as cEBRT, SBRT is better at delivering ablative doses (ranging from 7.7 Gy to 45 Gy in 1–4 fractions) while sparing vulnerable neurologic structures such as the spinal cord and nerve root [108]. Of note, SBRT also provides prolonged symptomatic relief and local tumor control of up to 90–95%, even in patients with radioresistant metastatic vertebral bone lesions, with or without prior irradiation [106,107,108,109]. Further, according to the largest multi-institutional cohort study of clinical practice and outcomes on spinal SBRT, this technique provides high clinical efficacy and can be used to treat several tumor types due to the absence of relative contraindications [110]. Nonetheless, further investigation is warranted to determine optimal dosing, fractioning, and the prospective long-term consequences of irradiation to neural structures.

Furthermore, a second and even more recent advancement in adjuvant therapies has been the utilization of image-guided percutaneous cryoablation in the treatment of neoplasms. Specifically, cryoablation, an already established and effective treatment method for liver and lung cancer, has recently emerged as a superior method in the treatment of metastatic bone cancer [111]. As mentioned above, this form of treatment entails placing percutaneous cryoprobes into affected areas and the cycling of freezing and thawing—which is achieved via the rapid expansion of argon gas, reaching temperatures lower than −40 °C in a matter of seconds, and the subsequent thawing through the infusion of helium gas [112]. Of note, this procedure is performed under image-guided assistance using intermittent contrast CT or MRI imaging technology. The aforementioned aspect of this technique is a significant advantage over heat-based techniques, such as radiofrequency ablation (RFA), because it allows surgeons to precisely monitor the ablative zone intraoperatively and thus confidently treat metacystic diseases more aggressively [113]. In conjunction with imagining, the introduction of catheter-guided balloons for tissue displacement is possible in percutaneous cryoablation, which has proven to be a large drawback of RFA due to thermal limitations [114]. Moreover, a final distinct advantage of cryoablation over other adjuvants is the reduction in post-procedural pain, as well as the ability to conduct the procedure with the patients under conscious sedation [115]. Regarding its clinical performance, several multicenter clinical trials have cited cryoablation to be safe and effective, with a majority of patients experiencing tumor reduction or arrest at follow-up, as well as a significant reduction in postoperative pain [116,117]. Although promising, this technology demands further investigation as its therapeutic effects have been limited to too few studies, in addition to a relative lack of prospective randomized studies comparing cryoablation to standard-of-care treatment options such as open surgery, chemotherapy, or radiation therapy [118].

## 5. Conclusions

Spine care professionals continue to be challenged by the complex nature of vertebral primary bone lesions. Indeed, given the abundance of lesion types, it is clear that further work comparing and contrasting different assessment and treatment strategies is needed. A more thorough analysis of different primary benign types, for example, could be warranted. Ultimately, however, despite their diversity, the overarching goal of any intervention for vertebral primary bone lesions is to restore patients’ quality of life and minimize post-treatment complications through the employment of the least invasive approaches available. Ideally, strategies should effectively treat both benign and malignant tumor types. Thus, it is clear that we must continue to develop novel interventions that will decrease invasiveness, increase clinical efficacy, and ultimately improve patient outcomes.

## Figures and Tables

**Table 1:** Common primary benign and malignant vertebral lesions and their associated radiographic findings on Magnetic Resonance Imaging (MRI) [29,32].

Primary Vertebral Lesion	Benign or Malignant	Radiographic Findings (MRI)
Spinal Meningioma	Benign	Well-circumscribed; Dural tail sign; broad-based Dural attachment
Osteoid Osteomas	Benign	Intracortical nidus; reactive sclerosis
Spinal Ependymomas	Malignant	Well-circumscribed; widened spinal cord; cystic change
Spinal Hemangioma	Benign	Variable enhancement; “salt and pepper” appearance
Chordomas	Malignant	Irregular calcification; honeycomb appearance
Ewing Sarcoma	Malignant	Bright with T2; not well-demarcated; non-homogenous enhancement of contrast
Nerve Sheath Tumors	Benign	Very well-circumscribed and difficult to identify on radiograph; hyperintense T2
Osteosarcoma	Malignant	Edema around tumor; cortical destruction; considerable contrast enhancement with T1
